# A novel approach to cancer rehabilitation: assessing the influence of exercise intervention on postoperative recovery and survival rates

**DOI:** 10.1097/JS9.0000000000002323

**Published:** 2025-03-28

**Authors:** Xiaoyan Chen, Zhi Li, Junfeng Zhang

**Affiliations:** a Medical College of Nanchang Institute of Technology, Nanchang, China; b Interventional Cancer Institute of Chinese Integrative Medicine, Putuo Hospital, Shanghai University of Traditional Chinese Medicine, Shanghai, China; c Rehabilitation Center of Taihe Hospital, Hubei University of Medicine, Hubei, China

**Keywords:** cancer rehabilitation, exercise intervention, postoperative recovery, postoperative rehabilitation, survival rates

## Abstract

Cancer rehabilitation is the crucial process by which cancer patients regain their physical abilities and enhance their quality of life through diverse methods following treatment. As the cure rate of cancer continues to rise, the need for postoperative rehabilitation is becoming increasingly evident. This is particularly crucial for enhancing patient survival rates and minimizing the chances of cancer recurrence. Exercise intervention has become increasingly popular and widely used as a proactive rehabilitation therapy in recent years. This article examines the influence of exercise intervention on the recovery and survival rates of cancer patients after surgery. It specifically investigates the effects and mechanisms of various exercise interventions, such as aerobic exercise, strength training, and flexibility training, on patients with lung cancer, gastric cancer, colorectal cancer, and other forms of cancer. Exercise therapies before and after surgery can greatly boost patients’ physical abilities, decrease postoperative problems, minimize hospital stays, and improve overall quality of life. In addition, implementing exercise intervention can enhance the long-term survival rates of patients. Future studies should investigate the most effective exercise programs and their suitability for various types of cancer, with the goal of offering better evidence-based clinical advice.

**HIGHLIGHTS**

- We reviewed the role of the exercise intervention in cancer rehabilitation.
- We examined potential exercise therapy for cancer rehabilitation.
- Future research should focus on enhancing the long-term effects of exercise therapy, particularly in terms of survival rates, recurrence rates, and quality of life.

## Introduction

Cancer is a prominent contributor to mortality and presents a significant risk to human well-being^[^1^]^. Cancer rehabilitation is a systematic approach that seeks to aid cancer patients in regaining physical abilities, enhancing mental health, and improving overall quality of life following surgery^[^2^]^. This is achieved by a combination of physiological, psychological, and social assistance. Postoperative rehabilitation extends beyond the restoration of physical function and includes a full recovery that involves psychological, social, and emotional components^[^3^]^. Rehabilitation training that follows a structured and organized approach is extremely important in enhancing the overall well-being and extending the lifespan of those diagnosed with cancer^[^4^]^. Rehabilitation training helps patients effectively deal with physical and psychological difficulties after surgery, improve their ability to function independently and their sense of self-worth^[^5^]^ and prevent long-term consequences, leading to a significant enhancement in their long-term quality of life^[^6^]^. Nevertheless, the rehabilitation process encounters obstacles such as inadequate medical resources and poor patient compliance, especially in places with minimal resources^[^7^]^. Furthermore, cancer patients frequently encounter different levels of psychological discomfort, such as anxiety and despair, following surgery, which might additionally affect their rehabilitation prospects^[^8^]^.

Exercise therapies have demonstrated considerable efficacy in enhancing the physical function and psychological well-being of individuals diagnosed with cancer^[^9^]^. Research has shown that exercise therapies can greatly improve physical function in individuals with cancer, leading to increased aerobic capacity, muscle strength, and flexibility^[^10^]^. Furthermore, physical activity can efficiently mitigate psychological anguish, such as anxiety and symptoms of depression, resulting in enhanced mental well-being and overall quality of life in individuals with cancer^[^11^]^. Nevertheless, the implementation of exercise therapies in cancer rehabilitation encounters obstacles, including the absence of studies examining long-term effects, standardized exercise prescriptions, and tailored advice.

This review assesses the impact of exercise therapies on postoperative recovery and survival rates in cancer patients, as well as their potential to improve physical function and mental health. Future research should explore the most effective exercise programs and their suitability for various forms of cancer. This will establish a theoretical foundation and offer practical recommendations for establishing tailored postoperative rehabilitation plans. To ensure that the literature review in this paper is comprehensive and representative, we employed a systematic literature retrieval approach. Specifically, we conducted searches in databases such as PubMed, Web of Science, and Scopus, using keywords including “physical activity,” “exercise,” “cancer patients,” and “quality of life” to cover various aspects related to the impact of physical activity interventions on postoperative recovery and survival rates in cancer patients. The inclusion criteria for the literature selection were as follows: we primarily selected peer-reviewed studies published within the past 5 years (2019–2024), prioritizing original research articles, systematic reviews, and meta-analyses, with a focus on studies involving cancer patients.

## Essential elements in the recovery process following cancer surgery

### Physiological factors

The physiological aspects that impact the recovery of cancer patients after surgery are vital in the rehabilitation process. These elements mainly involve the restoration of immune function and physical strength^[^12^]^. The immunological status following surgery is intricately linked to the clinical recuperation of patients. The signaling pathways of particular immune cells, such as CD14 + monocytes, are essential for the process of postoperative healing. These immune responses have the potential to be used as diagnostic indicators and targets for customized postoperative care^[^13^]^.

CD14 + monocytes, a type of specific immune cells, have a very important function in this process. CD14 + monocytes are a subset of white blood cells that belong to the mononuclear phagocyte system and carry out a range of tasks. These cells have the ability to prevent infections by identifying and engulfing harmful microorganisms^[^14^]^. They also have a key role in controlling inflammatory reactions by creating different substances that promote inflammation and by influencing the process of wound healing. The TLR4 signaling pathway in CD14 + monocytes plays a crucial role in beginning and controlling the immune response, guaranteeing a suitable inflammatory reaction, and facilitating swift recovery. Moderate physical activity can greatly improve the function of these cells by enhancing their circulation and activation, regulating inflammatory responses, boosting immune surveillance, and facilitating cell signaling^[^15^]^.These effects enhance the ability of CD14 + monocytes to actively contribute to immunological defence and tissue regeneration, leading to faster healing of postoperative wounds, decreased occurrence of complications, and ultimately promoting swift recovery and long-term well-being in patients. Exercise has a beneficial effect on postoperative immunomodulation, which helps maintain the stability and effectiveness of the immune system^[^16^]^.

The immunological status before surgery can be used to predict the recovery after surgery. The immune signal response prior to surgery is strongly correlated with the subsequent functional recovery and management of pain after surgery. Both the amount of physical activity before surgery and after surgery are significant indicators of how well a person would recover functionally after the operation. Greater preoperative physical activity is linked to quicker postoperative recovery, particularly in regards to the recovery of walking distance and leg strength^[^17^]^. Elderly patients with abdominal cancer exhibit noticeable short-term alterations in their physical recovery following surgery. Preoperative physical performance is correlated with improved postoperative functional ability and self-reported recovery^[^18^]^. The Enhanced Recovery After Surgery (ERAS) program has demonstrated notable efficacy in hastening clinical recovery and enhancing postoperative immunological function, particularly by lowering the duration of hospitalization after surgery and speeding the return of gastrointestinal function^[^19^]^. Postcancer surgery physiological recovery relies heavily on the restoration of immune function and physical strength. Targeting these factors through interventions can greatly enhance patients’ recovery process and overall quality of life. A comprehensive postoperative rehabilitation plan should incorporate individualized immune function assessment and suitable physical exercises to guarantee the best possible recovery results.

### Psychological recovery

Psychological variables are crucial in the recovery process of cancer patients, directly impacting the rate of postoperative recovery and overall quality of life^[^20^]^. Studies indicate that the preoperative psychological condition, including anxiety and depression, not only impacts the feeling of pain after cancer surgery but also has a significant correlation with the restoration of gastrointestinal function and the equilibrium of intestinal microbiota. For instance, preoperative anxiety and depression in breast cancer patients serve as indicators for postoperative pain^[^21^]^, whereas preoperative anxiety in colon cancer patients is linked to delayed recovery of gastrointestinal function and disruption in intestinal microbiota^[^22^]^. Hence, preoperative psychological preparation is vital, as it can effectively diminish surgical discomfort, expedite behavioral recuperation, and decrease the incidence of adverse feelings.

Psychological therapies are highly influential in this process. These interventions have the ability to significantly decrease anxiety and depression levels in rectal cancer patients who are receiving chemotherapy, promote immune system functioning, and improve adherence to treatment^[^23^]^. High-quality psychological intervention therapy for breast cancer patients has a beneficial influence on their mental health, significantly decreasing postoperative problems and enhancing patient satisfaction^[^24^]^. Integrating psychological interventions with traditional Chinese medicine treatments can enhance the reduction of psychological stress response and inflammatory indicators in patients with advanced cervical cancer, leading to improvements in both quality of life and survival rates^[^25^]^. The recovery process can be significantly improved by effectively controlling postoperative sadness and anxiety, as emotional stability plays a vital role in clinical recovery^[^26^]^. Engaging in active psychological interventions can greatly enhance the quality of life for patients, alleviate postoperative physical discomfort and psychological distress, and play a crucial role in promoting total recovery and well-being^[^27^]^.

Recent research has demonstrated that exercise treatment has a substantial enhancing impact on psychological aspects. Physical activity has the ability to quickly, securely, and efficiently enhance the mental well-being of those suffering from depression^[^28^]^. A comprehensive analysis revealed that exercise treatment had substantial impacts on enhancing attention, cognitive capabilities, and overall quality of life. Furthermore, it exerts a beneficial influence on the social conduct of children and adolescents who have autism and learning disabilities^[^29^]^. Physical exercise can greatly improve the overall quality of life, energy levels, and mental health of persons who are fat or overweight^[^30^]^. The combination of suitable high-intensity exercise and behavioral therapy has been shown to effectively reduce symptoms of depression and anxiety^[^31^]^. Furthermore, exercise can function as a supplementary method to medication and psychological therapy, assisting in the enhancement of symptoms related to significant mental illnesses^[^32^]^. Physical activity therapy is essential for promoting mental well-being by effectively reducing symptoms of depression and anxiety, hence enhancing general psychological health and quality of life.

## Categories of physical activity interventions

### Aerobic exercise

Aerobic exercise is a form of physical activity that enhances cardiovascular function and metabolic capacity by engaging in sustained activity for an extended duration^[^33^]^. The primary features of this include a moderate level of intensity and a protracted period of time. Typical examples of aerobic exercises are fast-paced walking, jogging, swimming, and cycling. These activities increase heart rate and breathing, which improves the intake and use of oxygen, leading to better cardiovascular function, lower blood pressure and cholesterol levels, and improved cardiovascular health.

Aerobic exercise is essential for recovering from cancer surgery, since numerous studies have shown that it has a beneficial impact on improving patients’ physical and mental health. Aerobic exercise not only boosts the physical fitness of individuals with cancer, but also improves their quality of life, reduces the duration of hospitalization, and aids in the prevention of postoperative complications^[^34^]^. A meta-analysis revealed that cancer patients who participated in aerobic exercise during their postoperative recovery exhibited enhanced cardiopulmonary function, reduced hospitalization duration, and decreased incidence of postoperative complications in comparison to those who did not partake in aerobic exercise. In addition, these individuals also shown a substantial enhancement in their quality of life scores^[^35^]^.

The combination of aerobic exercise with *Tai Chi* in lung cancer patients yields notable enhancements in sleep quality, reductions in fatigue, and decreases in levels of worry and sadness. *Tai Chi* (also spelled Taiji or Tai Chi Chuan), is a traditional Chinese martial art that combines slow, deliberate movements, meditation, and deep breathing^[^36^]^. It is rooted in the principles of Yin and Yang, emphasizing balance, fluidity, and harmony between the mind and body. *Tai Chi* is practiced for its potential health benefits, including improved flexibility, strength, mental clarity, and stress reduction. It is also considered a form of “moving meditation” that promotes inner peace and well-being. These findings indicate that the simultaneous utilization of aerobic exercise and mind-body techniques such as *Tai Chi* can significantly improve the overall health condition of individuals with cancer^[^37^]^. A systematic review and meta-analysis have shown that aerobic training can greatly increase patients’ aerobic capacity, as indicated by a considerable enhancement in aerobic capacity during the 6-min walk test^[^35^]^. A randomized controlled research discovered that a brief, home-based aerobic exercise intervention has a positive effect on the recuperation of patients following colorectal cancer surgery. This intervention demonstrates the practicality of including exercise while assuring safety and the absence of any negative occurrences^[^38^]^. A meta-analysis of nonsmall cell lung cancer patients suggests that incorporating preoperative aerobic exercise, along with other exercise modalities, can yield substantial enhancements in walking endurance, maximum exercise capacity, and a reduction in postoperative complications^[^39^]^. Engaging in high-intensity interval aerobic exercise before surgery can improve the cardiopulmonary function of patients with bladder cancer. Nevertheless, additional investigation is required to ascertain its precise influence on postoperative recuperation^[^40^]^. The latest research findings underscore the significance of aerobic exercise during the full course of cancer treatment. It not only enhances postoperative recuperation but also offers assistance throughout therapy, improving patients’ physical and psychological well-being.

### Strength training

Strength training is a form of physical activity that improves muscle strength and endurance by using resistance training. The main objective is to induce muscle contraction through repetitive resistance against external forces (such as weights, resistance bands, etc.), resulting in enhanced muscle growth, strength, and functionality^[^41^]^. Popular strength training techniques encompass weightlifting, utilization of fitness apparatus, resistance band exercises, and bodyweight movements like push-ups and squats. Strength training not only increases muscle strength and size, but also raises bone density, therefore preventing osteoporosis. A study on strength training intervention for post-cancer surgery recovery suggests that strength training has a beneficial effect on postoperative rehabilitation^[^42^]^. Colorectal cancer patients who engage in strength training both before and after surgery experience notable enhancements in their cardiorespiratory function and physical recovery, in contrast to individuals who do not participate in training^[^38^]^. Strength training had a notable positive impact on the strength of muscles in the upper and lower limbs, as well as improving the quality of muscle tissue in individuals with breast cancer^[^43^]^. Customized resistance training has the potential to enhance the overall well-being and physical abilities of those who have undergone cancer surgery^[^44^]^. Furthermore, studies have shown that it significantly reduces fatigue in cancer patients, leading to significant improvements in their functional and activity levels^[^45^]^.

Strength training has the potential to greatly enhance the balance and postural stability of cancer patients who are receiving chemotherapy, hence decreasing the likelihood of falls^[^46^]^. Studies have shown that practicing breathing exercises to strengthen the muscles involved in inhaling during the 2 weeks following surgery can greatly enhance the oxygen levels in lung cancer patients who are at a higher risk^[^47^]^. A systematic review has found that engaging in resistance training before and after abdominal surgery can have a beneficial effect on the physical function recovery of cancer patients^[^48^]^. Additionally, studies have shown that an exercise program that leverages internet and social media platforms significantly enhances both quality of life and muscle strength^[^49^]^. Strength training has a beneficial impact on postcancer surgery recovery and also improves fatigue, physical activity, muscle strength, and functional capacity during the early recovery after hematopoietic stem cell transplantation^[^50^]^. It effectively enhances patients’ muscle strength, endurance, cardiovascular function, and quality of life, while reducing fatigue.

### Flexibility training

Flexibility training is an exercise method that focuses on enhancing the range of motion in joints and increasing muscular flexibility by doing stretching and movement exercises. The main objective of this activity is to enhance the body’s flexibility and agility by elongating muscles and joints through deliberate and measured movements^[^51^]^. Typical forms of flexibility training encompass yoga, Pilates, and a range of stretching activities. Flexibility exercise can improve joint mobility, relieve muscular stress and stiffness, and help prevent sports injuries. Flexibility training enhances body balance and coordination, thereby enhancing overall athletic performance^[^52^]^.

Additionally, it can substantially alleviate the discomfort encountered by cancer patients receiving chemotherapy, thereby enhancing their quality of life^[^53^]^. Preoperative and postoperative flexibility training, together with other types of exercise, had a considerable positive impact on the physical recovery of patients undergoing colon cancer surgery^[^38^]^. Pilates training has been found to have a considerable positive impact on muscle strength and flexibility in the lower and upper limbs of breast cancer patients after surgery^[^54^]^. Among cancer patients receiving chemotherapy, the integration of flexibility training with multi-component training yielded notable enhancements in postural stability and joint range of motion. Additionally, it exerted a regulatory influence on blood pressure^[^55^]^. Research has demonstrated that early and comprehensive exercise programs, encompassing aerobic exercise, strength training, and flexibility training, significantly improve the physical function of cancer patients undergoing abdominal surgery^[^56^]^. Resistance training before and after surgery has a beneficial effect on the physical function recovery of cancer patients who are having abdominal surgery^[^48^]^. Flexibility training has the potential to decrease symptoms, promote physical function and flexibility, improve physical recovery and postural stability, ultimately leading to an improvement in overall sports performance and quality of life.

### Other modalities of physiotherapy

Aside from the three fundamental categories of exercise therapy stated earlier, there are other specialized forms of exercise therapy that possess distinct applicability in certain medical and health domains. Studies have demonstrated that *Tai Chi*, when used as a complementary treatment, can greatly enhance lung function, oxygen intake, and quality of life in patients who have undergone surgery for nonsmall cell lung cancer^[^57^]^. Additionally, there is a notable improvement in the psychological and physiological symptoms of patients with breast cancer^[^58^]^. Scientific studies and analyses show that engaging in high-intensity interval training (HIIT) before surgery can greatly enhance the cardiorespiratory function of individuals with cancer^[^59^]^. While there is a lack of particular research on the use of aquatic exercise therapy for postcancer surgery recovery, studies have demonstrated that other forms of aerobic exercise, such as walking and cycling, can effectively reduce fatigue during cancer treatment and enhance overall quality of life^[^60^]^. Yoga intervention has been shown to have a substantial impact on reducing the length of postoperative hospital stay, the duration of drainage tube retention, and the time required for suture removal in breast cancer patients. Additionally, it has the ability to reduce the concentration of tumor necrosis factor (TNF) in the blood plasma, which can assist in the process of recovering after surgery^[^61^]^.

## The effects of exercise intervention on the recovery and survival rates after surgery

### Effect on immediate postoperative recovery

Physical intervention is a highly successful approach to greatly improve patients’ immediate recovery after surgery. It helps to alleviate pain, shorten the recovery period, and promote functional recovery^[^62^]^. Through the implementation of individualized exercise regimens, patients can efficiently control pain and decrease dependence on medication. Exercise interventions enhance blood circulation, hasten the healing of injured tissues, and hence speed the recovery process^[^63^]^. In addition, engaging in physical exercise can boost patients’ muscular strength and joint flexibility, increase their mental state, decrease the likelihood of postoperative sadness and anxiety, and improve their overall quality of life^[^64^]^.

Several studies have investigated the effects of exercise intervention after surgery on short-term healing. Preoperative exercise intervention for rectal cancer surgery has been shown to greatly improve patients’ physical recovery^[^38^]^. Researchers have found that a 4-week at-home exercise program, including aerobic activity and training of breathing muscles, improves the physical condition of patients with colorectal cancer postsurgery^[^38^]^. Studies suggest that resistance training can have a beneficial effect on the rehabilitation of physical function in individuals with abdominal cancer after surgery^[^48^]^. A meta-analysis of preoperative exercise therapy for gastrointestinal cancer patients discovered that engaging in aerobic exercise prior to surgery can enhance patients’ cardiopulmonary function and expedite the recovery of their physical abilities after the operation^[^65^]^. A study examining the effects of exercise intervention on postoperative lung cancer patients demonstrated a considerable improvement in both patients’ exercise ability and health-related quality of life^[^66^]^. Exercise training after surgery can successfully enhance the physical function recovery of lung cancer patients who also have chronic obstructive pulmonary disease^[^67^]^. A study examining the effects of a home exercise program on breast cancer patients found that engaging in a 1-year exercise plan can greatly enhance shoulder muscle strength and shoulder joint mobility, while simultaneously reducing shoulder pain and disability scores^[^68^]^. Exercise intervention plays a vital role in the recovery after surgery, not only improving patients’ physical health but also contributing to their psychological well-being. This leads to a more effective and thorough rehabilitation process.

### Effect on long-term viability

Research has investigated the effects of exercise therapies after surgery on the long-term survival rates of individuals with cancer. Studies have shown that engaging in exercise both before and after surgery can improve the overall well-being and reduce fatigue levels in patients who have undergone colorectal and lung cancer surgeries^[^69^]^. A study conducted on breast cancer patients has discovered that exercise interventions with remote direction can greatly enhance patients’ quality of life, muscle strength, and cardiovascular endurance^[^70^]^. Further investigation is required in the future to establish the correlation between physical activity and the occurrence of uncommon cancers, as well as the connection with the long-term rates of survival for other cancers. Future research on cancer occurrence and death rates should take into account the importance to specific groups within the community, establish the correlation between physical activity levels and the risk and prognosis of cancer, and clarify the processes that explain these connections^[^71^]^. While some studies have indicated that exercise therapies can enhance the survival rate of individuals with cancer, the existing research is insufficient, and additional large-scale cohort studies are required to delve deeper into this matter.

## Biological mechanisms of exercise therapy

### Regulation of the inflammatory reaction

The process by which exercise regulates the body’s inflammatory response involves various physiological and biochemical pathways. Its main effect is to decrease the levels of pro-inflammatory cytokines [such as IL-6, tumor necrosis factor-alpha (TNF-α), and C-reactive protein (CRP)] and increase the expression of anti-inflammatory cytokines (such as IL-10)^[^72^]^. Cytokines have a vital function in the inflammatory response, and excessive production of these molecules is strongly linked to the emergence of several chronic illnesses and malignancies. A study investigating the effects of early exercise on neuroinflammatory pathways and emotional disorders in a rat model of stroke discovered that regular exercise can decrease the likelihood of developing chronic metabolic and cardiovascular diseases, as well as the occurrence of stroke. Furthermore, it has the ability to reduce low-grade inflammatory reactions and mental disorders^[^73^]^. Suppressing pro-inflammatory cytokines hampers the production and release of inflammatory cytokines, hence diminishing the magnitude and extent of inflammatory reactions. In addition, physical activity reduces body fat and enhances the metabolism of adipose tissue, resulting in a decrease in the production of pro-inflammatory substances by fat cells and immune cells. This, in turn, helps reduce inflammation in adipose tissue. Exercise stimulates the AMPK and PPAR-γ pathways in cells, which increases the breakdown of fatty acids and uptake of glucose. It also decreases the production of inflammatory substances and enhances the activity of antioxidant enzymes like SOD and GPx, thus reducing inflammation caused by oxidative stress^[^74^]^.

Exercise stimulates metabolic changes, such as enhanced uptake and utilization of glucose in skeletal muscle tissue, as well as the synthesis of bioactive molecules like IL-6, which are important for regulating energy metabolism. IL-6 stimulates the breakdown of fats in adipose tissue and enhances the body’s ability to use insulin, hence promoting energy metabolism during exercise^[^75^]^. During exercise, it increases the generation of glucose in the liver and the breakdown of fat in adipose tissue. This helps to meet the increased need for glucose in skeletal muscle tissue during physical activity^[^76^]^. Several studies have demonstrated that exercise can regulate inflammatory responses through several mechanisms, therefore enhancing the process of recovering after surgery. Intense physical activity can greatly decrease the levels of CRP and TNF-α in breast cancer patients who have undergone chemotherapy. This helps to relieve inflammation caused by chemotherapy^[^77^]^. Aerobic and resistance exercise programs can effectively decrease systemic inflammation levels in obese and sedentary cancer survivors. This includes reducing the concentrations of IL-6, IL-8, and TNF-α^[^78^]^. Research suggests that preoperative and postoperative resistance training can have different impacts on cancer patients who are undergoing abdominal surgery. However, it has been found that this type of exercise can improve postoperative physical function and promote the body’s inflammatory response^[^48^]^. Studies have demonstrated that implementing a preoperative rehabilitation exercise program in patients with lung cancer leads to a notable decrease in postoperative problems, a shorter hospital stay, and enhanced recovery after surgery. This is achieved by increasing the function of the heart and lungs and reducing inflammatory responses^[^79^]^.

**Figure 1.**
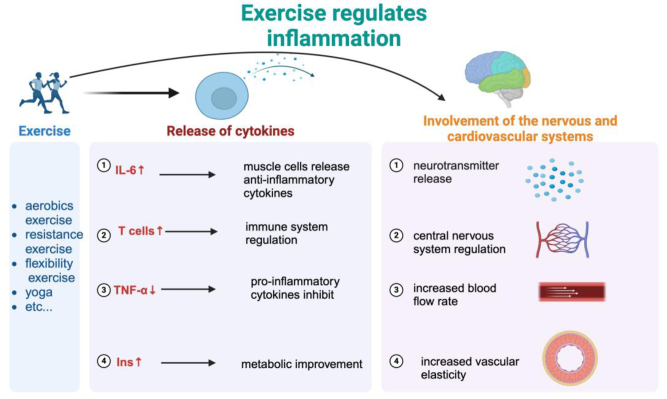
Physical activity exerts regulatory effects on the immune system, diminishing inflammation, optimizing blood circulation, augmenting tissue oxygenation, and fostering general well-being through the release of anti-inflammatory substances and the inhibition of pro-inflammatory synthesis. *Created with BioRender.Com.*

Exercise is essential in decreasing postoperative inflammation in cancer patients by regulating multiple levels and pathways. These systems not only assist in the recuperation of individuals with cancer but also have important implications for the prevention and control of other long-lasting inflammatory illnesses (Fig. 1).

### Modulation of hormone levels

Physical activity has the potential to greatly enhance the body’s ability to respond to insulin, decrease the body’s resistance to insulin, regulate blood sugar levels by enhancing the absorption and utilization of glucose by muscle cells, and stimulate the production of glycogen in the liver to counteract the inflammatory consequences of high insulin levels^[^80^]^. Physical activity can stimulate the release of endorphins and dopamine, enhance emotional well-being, and reduce symptoms of anxiety and depression^[^81^]^. Exercise has been found to decrease adipose tissue and regulate aromatase activity in female breast cancer patients, leading to a reduction in estrogen levels and a decreased risk of breast cancer recurrence^[^82^]^. In male prostate cancer patients, engaging in physical activity can increase testosterone levels, improve physical strength, and enhance quality of life^[^83^]^.

In addition, moderate exercise helps to control cortisol levels, mitigate stress responses, and lower cortisol levels during periods of rest^[^84^]^. Physical activity triggers the release of growth hormone and insulin-like growth factor, which enhance cellular growth, repair, and metabolic control^[^85^]^. Combining food intervention with exercise treatment has been proven to positively affect the quality of life and psychological well-being of breast cancer patients. This may be due, at least in part, to alterations in hormone levels^[^86^]^. Furthermore, therapies that include both endurance and strength training have the ability to lower levels of inflammatory markers, such as CRP and TNF-α. Hormone-level adjustments may be responsible for this reduction in indicators^[^77^]^. A separate study has shown that implementing exercise can effectively decrease the levels of systemic inflammation in cancer survivors who are both obese and inactive. This includes reducing the concentrations of IL-6, IL-8, and TNF-α, which also indicates alterations in hormone levels^[^87^]^.

**Figure 2.**
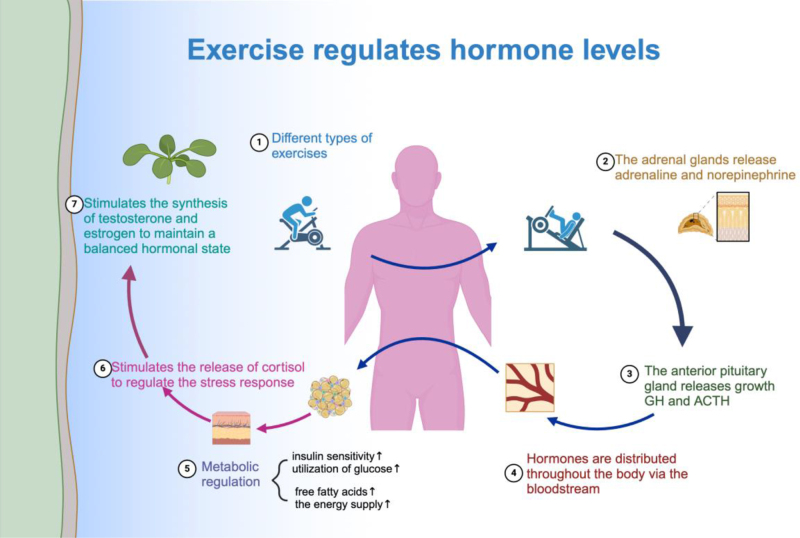
Physical activity maintains hormone levels, improving energy metabolism, mood, immunity, and regulating stress hormones. It promotes physiological balance and mental well-being by stimulating the endocrine system. *Created with BioRender.Com.*

Engaging in physical activity enhances the metabolic status and emotional well-being of individuals with cancer by regulating crucial hormone levels, therefore facilitating their recovery and overall health (Fig. 2).

### Enhancing immunological function

Exercise improves the immune system by increasing the activity of immune cells, elevating levels of immunoglobulins, enhancing blood circulation and lymphatic movement, stimulating the HPA axis and release of adrenaline, and lowering chronic inflammation, among other mechanisms^[^88^]^. Physical activity can boost the functioning of natural killer (NK) cells and T cells, elevate the levels of immunoglobulins in saliva and blood, accelerate cardiac output and lymphatic flow, therefore enhancing the effectiveness of immune cell distribution^[^89^]^. Exercise stimulates the release of adrenaline and noradrenaline by activating the hypothalamic–pituitary–adrenal (HPA) axis, which strengthens the immune system’s stress response^[^90^]^. In addition, engaging in moderate physical activity can help decrease chronic inflammation by regulating the levels of substances that promote inflammatory conditions, reducing the production of molecules that promote inflammation, and increasing the production of molecules that reduce inflammation. This can help alleviate the negative impact of chronic inflammation on the immune system.

Exercise has significant impacts on the functioning of the immune system. A single session of exercise triggers a temporary immune response, which, when repeated over time, results in the immune system’s ability to adapt to chronic exercise training. The impact of exercise on immunity varies depending on the intensity and duration. Prolonged high-intensity exercise training can weaken the immune system, whereas regular exercise training can stimulate thymus activity and improve overall immune function by reducing inflammation. This ultimately lowers the likelihood of diseases like upper respiratory tract infections. Research has demonstrated that exercise interventions can decrease the likelihood of cancer returning or the development of further cancerous growths by improving immune system reactions and prolonging survival duration^[^91^]^. The exercise program, when paired with nutritional management, has a beneficial effect on the immune system of individuals with breast cancer^[^86^]^. Respiratory function training has a considerable effect on the immune function and occurrence of pulmonary infections after surgery in individuals with lung cancer. The cohort that received respiratory function training exhibited substantial enhancements in postoperative immune function, characterized by notable decreases in TNF-α, IL-8, and IL-6 concentrations, together with a considerable rise in CD4 + count and CD4 +/CD8 + ratio^[^92^]^. A systematic analysis demonstrates that implementing exercise intervention before to surgery can effectively decrease postoperative problems in patients with lung cancer, as well as enhance postoperative lung function and immunological function in this patient population^[^93^]^. The effects of resistance training before and after abdominal surgery in cancer patients have been found to be inconsistent. This training has the potential to improve postoperative physical performance and promote immunological response^[^48^]^. The administration of Preoperative Immune Modulating Nutrition (IMN) has been shown to effectively decrease postoperative infection problems and minimize hospitalization duration, maybe as a result of enhancing the immune system^[^94^]^. A study has demonstrated that physical exercise intervention can improve the activity of NK cells in breast cancer patients after surgery. Specifically, engaging in moderate-intensity exercise early on has been found to have positive impacts on the function of NK cells^[^95^]^.

**Figure 3.**
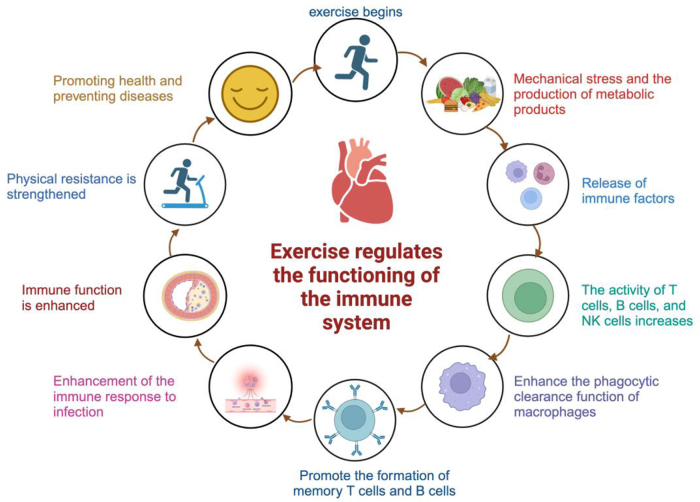
Engaging in physical activity enhances the functioning of the immune system, increases the body’s ability to resist diseases, and reduces the likelihood of getting infected. It enhances blood circulation, lymphatic flow, and waste removal, so assuring overall health and immune function. *Created with BioRender.Com.*

The exercise intervention after cancer surgery seeks to improve the performance of the immune system, decrease inflammatory factors, enhance NK cell activity, and optimize the ratio of immune cells. This intervention is designed to promote a faster recovery after the surgery (Fig. 3).

## Implementation strategy of exercise therapy

### Develop individualized exercise plan

The exercise plan is tailored to an individual’s health status, physical fitness level, and exercise goals in order to provide specific recommendations about the frequency, intensity, duration, and type of exercise. The objective of this strategy is to support individuals in participating in organized and regular exercise, guaranteeing thorough physical conditioning to enhance general well-being and mental calmness. The formulation of a customized exercise regimen considers the unique requirements of each individual, guaranteeing that every person can select the suitable exercise modality according to their own circumstances, in order to attain the most favorable outcomes from their workout sessions. An individualized exercise regimen not only guarantees the safety of the workout, preventing health hazards resulting from inappropriate exercise intensity, but also customizes the most efficient exercise techniques and frequencies to suit specific personal objectives (such as weight reduction, muscle strengthening, and cardiovascular improvement), thereby enhancing the efficacy of the exercise and boosting patients’ commitment to regular physical activity.

In order to create tailored exercise regimens, it is necessary to thoroughly take into account several criteria such the individual’s age, gender, physical condition, previous exercise routines, body mass index (BMI), history of chronic illnesses, and psychological well-being. Engaging in exercise before surgery can greatly improve lung function, shorten the length of hospitalization, and lower the chances of experiencing difficulties after the operation. Prior to surgery, it is advisable to incorporate a 2-4 week regimen of aerobic and strength training^[^96^]^. Another study indicates that implementing exercise intervention before surgery might substantially decrease the occurrence of postoperative problems in individuals with lung cancer, as well as minimize the duration of hospitalization. Highlighting individualized workout regimens plays a pivotal function in enhancing exercise engagement and efficacy^[^97^]^. A systematic review suggests that engaging in resistance training before and after surgery can improve postoperative physical function and immunological response. It is recommended to perform moderate-intensity aerobic and resistance training 2–4 weeks before to the surgery^[^48^]^.

Participating in suitable physical activities might significantly increase compliance with postoperative exercise among breast cancer patients, facilitating the recovery of impaired limb function and the enhancement of self-care skills^[^98^]^. Engaging in exercise before surgery can improve heart and lung function and speed up the recovery process after surgery. It is advisable to use a multimodal exercise intervention program that includes both aerobic and strength training^[^65^]^. Exercise therapies before and after surgery can greatly improve walking endurance, maximum exercise capacity, and decrease the length of hospital stay and postoperative pulmonary complications^[^39^]^. It is recommended that patients who are new to exercise start with low-intensity aerobic activities and gradually increase both the length and intensity of their workouts^[^99^]^. Patients who are already physically fit can benefit from combining strength training with high-intensity interval training (HIIT)^[^100^]^. It is crucial to take into account the patient’s psychological condition, and it is recommended to include stress-reducing activities like yoga and *Tai Chi*^[^101^]^. The creation of tailored exercise plans is a meticulous procedure that considers both the physical and psychological requirements of patients. It is also crucial for guaranteeing the safety and efficacy of exercise.

### Create a treatment strategy tailored to the specific type and stage of the tumor

The exercise regimen should be customized according to the patient’s specific tumor classification, stage of disease, and ongoing treatment protocol. Various tumor forms have diverse effects on the physical fitness of patients. For instance, individuals with breast cancer may need targeted exercises for the upper body to improve muscle strength and flexibility^[^102^]^. On the other hand, patients with colorectal cancer should focus on recovering their abdominal muscles^[^103^]^. Treatment plans should be adjusted according to the different stages of cancer, with early-stage patients being able to handle more intense exercise^[^104^]^. In contrast, individuals in advanced stages may benefit from gentle exercises like yoga and stretching to maintain physical strength^[^60^]^.

Exercise prescriptions are personalized based on factors such as age, health condition, and personal preferences, effectively managing symptoms like fatigue and pain. Engaging in suitable physical activity not only aids in preserving physical functionality during treatment and minimizing adverse effects, but also facilitates patients in resuming their regular daily routine post-treatment. Prolonged adherence may additionally decrease the likelihood of cancer relapse and enhance rates of survival. Preoperative and postoperative exercise therapies have been shown to have a substantial impact on patients with nonsmall cell lung cancer. These interventions can greatly enhance walking endurance, maximal exercise capacity, and lead to a reduction in hospital stay and postoperative pulmonary problems. Prior to surgery, it is advisable to engage in aerobic, resistance, and inspiratory muscle training for a period of 2–4 weeks^[^39^]^. Preoperative exercise intervention can enhance cardiopulmonary function and expedite postoperative recovery in patients diagnosed with gastrointestinal cancer. It is advisable to employ a multimodal exercise intervention strategy, which involves both aerobic training and strength training^[^65^]^.

Resistance training before and after surgery for individuals with stomach cancer can improve physical function and immunological response after the operation. Studies suggest that it is advisable to include moderate-intensity aerobic and resistance training prior to undergoing surgery^[^48^]^. Aerobic exercise can boost compliance with postoperative functional exercise in breast cancer patients, leading to improved restoration of physical function and self-care abilities^[^17^]^. Tailored exercise treatment programs, designed according to the specific characteristics and progression of the tumor, can greatly improve the results of rehabilitation and the overall well-being of patients (Table 1).

**Table 1:** Exercise therapy strategies for various tumor types

Types of tumor	Types of exercise	Exercise frequency	Exercise intensity	Exercise duration	Exercise effectiveness
Breast cancer^[^105^]^	Aerobic exercise	3–5 times per week	Moderate intensity	Each session lasts 30–60 min	Improve physical fitness; reduce fatigue
Colorectal cancer^[^106^]^	Strength training combined with aerobic exercise	3–4 times per week	Moderate intensity	Each session lasts 40–60 min	Improve physiological functions; enhance quality of life
Lung cancer^[^37^]^	Aerobic exercise	3–5 times per week	Moderate intensity	Each session lasts 30–45 min	Reduce respiratory distress; enhance endurance
Prostate cancer^[^83^]^	Strength training and flexibility training	2–3 times per week	Low to moderate intensity	Each session lasts 20–40 min	Enhance muscle strength; improve muscle. function
Stomach cancer^[^107^]^	Aerobic exercise	3–5 times per week	Low to moderate intensity	Each session lasts 20 min	Reduce postoperative fatigue
Ovarian cancer^[^108^]^	Low-intensity aerobic exercise	3–4 times per week	Low intensity	Each session lasts 20–30 min	Promote mental well-being; alleviate anxiety

### Potential exercise therapy for cancer rehabilitation

Throughout the postcancer surgery recovery period, alongside conventional aerobic exercise, strength training, and flexibility training, some alternative therapies have demonstrated potential efficacy. Aquatic exercise is appropriate for cancer rehabilitation patients who have weakened physical conditions because it is low-impact and provides mild support for joints. This type of exercise can enhance patients’ cardiopulmonary function and improve their quality of life^[^109,110^]^. Yoga, through the combination of physical postures, breath control, and meditation, effectively reduces anxiety, exhaustion, and sleep difficulties in patients^[^111-113^]^. *Tai Chi* and dance therapy share the common goal of enhancing physical function and psychological well-being in older people through the use of gentle motions and rhythmic exercises^[^114^]^. Intermittent high-intensity training, despite its intensity, is particularly efficient in enhancing cardiovascular endurance^[^115,116^]^.

Furthermore, it is worth considering other exercise therapies that are used in the rehabilitation of different illnesses, including functional training, neuromuscular electrical stimulation (NMES), balance training, sports activities combined with cognitive behavioral therapy, and autonomic nervous system training^[^117,118,118,119^]^. These therapies may offer additional recovery options for cancer patients. Novel treatments like golf and therapeutic cycling offer low-intensity physical exercise that fosters social engagement and mental relaxation^[^120,121^]^. Incorporating therapeutic games like Wii Fit or virtual reality games adds an enjoyable and dynamic element to rehabilitation, motivating patients to actively participate in their recovery activities^[^122,123^]^. Engaging in activities like rock climbing therapy and martial arts forms like Aikido, under controlled conditions, can greatly improve patients’ physical strength, flexibility, and balance^[^124-127^]^. Our objective is to improve the quality of life for cancer patients and expedite their recovery by employing a wide array of rehabilitation treatments (Table 2).

**Table 2:** Potential exercise therapy for cancer rehabilitation.

Exercise therapy	Features and benefits	References
Aquatic exercise	Low-impact exercise, suitable for cancer patients in weak physical condition, improves cardiovascular function and quality of life.	^[^109,110^]^
Yoga	By integrating body posture, breath control, and meditation, alleviate anxiety, fatigue, and sleep disorders.	^[^111,112^]^
*Tai Chi* and dance therapy	Gentle exercise, aids in enhancing physical function and psychological well-being in elderly patients.	^[^128,129^]^
Intermittent high-intensity training	Despite its high intensity, it can effectively improve cardiovascular endurance.	^[^115,116^]^
Functional training	Enhance daily life activity skills.	^[^117,118,118,119^]^
NMES	Improve muscle function to facilitate rehabilitation.	^[^117,118,118,119^]^
Balance training	Improve balance capabilities to reduce the risk of falling.	^[^117,118,118,119^]^
Sports activities incorporating cognitive behavioral therapy	Enhance both mental well-being and physical capabilities.	^[^117,118,118,119^]^
Autonomic nervous system training	Facilitate self-regulation and relaxation.	^[^117,118,118,119^]^
Golf and therapeutic cycling	Provide gentle physical activities to promote social interaction and mental relaxation.	^[^120,121^]^
Gaming therapy (such as WiiFit or VR games)	Enhance the fun and dynamic nature of rehabilitation activities to increase engagement.	^[^122,123^]^
Rock climbing therapy and martial arts forms such as Aikido	Significantly enhance physical strength, flexibility, and balance in a controlled environment to improve physical fitness and mental resilience.	^[^124-127^]^

## Challenges and future research directions

### Limitations of the current research

Presently, the predominant emphasis in cancer rehabilitation research on exercise treatment lies in investigating its immediate impacts, such as ameliorating fatigue, augmenting quality of life, and promoting physical fitness. Nevertheless, there is a dearth of extensive, enduring randomized controlled studies that investigate the long-term consequences of this treatment, such as its influence on survival rates, recurrence rates, and long-term quality of life. This constraint impedes a thorough comprehension of the long-lasting advantages that exercise therapy may provide in cancer rehabilitation, thereby impacting its promotion and implementation in clinical practice.

Moreover, there is a lack of extensive study regarding the disparities among different types of cancer and the specific groups of patients affected. Although studies have confirmed the rehabilitative effects of certain cancer types like breast cancer and colorectal cancer, there is a relative scarcity of research on other types of cancer such as pancreatic cancer and liver cancer, as well as specific patient populations including elderly patients, pediatric patients, and patients with multiple comorbidities. The lack of study in this area hinders the development of tailored and accurate rehabilitation programs for cancer patients with various types and conditions, thereby limiting the full usage of exercise therapy in cancer rehabilitation.

### Prospective area of investigation

Future cancer recovery research should prioritize investigating the enduring advantages of exercise treatment by examining its impact on survival rates, recurrence rates, and quality of life. This should be done through large-scale randomized controlled trials to validate its long-term effects. Moreover, it is imperative to augment research on the impact of exercise treatment on various cancer types and patient cohorts (including the elderly, children, and individuals with multiple comorbidities) to facilitate the development of tailored exercise regimens. Furthermore, it is necessary to conduct a comprehensive examination of the biological processes involved in exercise therapy. This should involve the use of advanced molecular biology techniques to uncover the impact of exercise on the growth and spread of cancer cells, as well as its effects on immune function.

Additionally, it is important to investigate how exercise influences the surrounding environment of tumors and their metabolic properties. The integration of knowledge from several domains, such as exercise science, oncology, psychology, and nutrition, is crucial for interdisciplinary collaboration in order to create comprehensive rehabilitation programs. Furthermore, the utilization of wearable devices and mobile applications enables the continuous monitoring of a patient’s health state in real-time. This allows for the provision of individualized exercise recommendations, which in turn improves exercise compliance and efficacy.

## Conclusion

Extensive research has shown that exercise treatment after cancer surgery is highly effective in improving patients’ physical function, enhancing their quality of life, and reducing problems after the surgery. Future research should focus on enhancing the long-term effects of exercise therapy, particularly in terms of survival rates, recurrence rates, and quality of life. Additionally, there is a need to conduct personalized studies on various types of cancer and different patient populations. It is important to delve into the biological mechanisms underlying exercise therapy and promote interdisciplinary collaboration. Furthermore, comprehensive rehabilitation research should be conducted by integrating knowledge from relevant fields. Lastly, the use of wearable devices and mobile applications should be explored to improve patients’ adherence to exercise and its effectiveness. By investigating these research avenues, the efficacy of exercise treatment in cancer rehabilitation can be significantly enhanced, hence improving the long-term health and quality of life of patients.

## Data Availability

None.
